# Complete genome sequence of *Blautia luti* JCM 17040^T^

**DOI:** 10.1128/MRA.00513-23

**Published:** 2023-08-31

**Authors:** Hironori Fukuoka, Dieter M. Tourlousse, Mayu Hamajima, Kazuyoshi Koike, Itaru Endo, Yuji Sekiguchi

**Affiliations:** 1 Department of Gastroenterological Surgery, Yokohama City University, Kanagawa, Japan; 2 Biomedical Research Institute, National Institute of Advanced Industrial Science and Technology (AIST), Tsukuba, Ibaraki, Japan; Wellesley College Department of Biological Sciences, Wellesley, Massachusetts, USA

**Keywords:** human gut microbiota, type strain, genome sequence, *Blautia luti*

## Abstract

We generated a complete genome sequence of the type strain of *Blautia luti* (JCM 17040^T^ = DSM 14534^T^) by Nanopore sequencing. The genome consists of a circular chromosome of 3,741,599 bp with a G + C content of 42.9% and was predicted to contain 3,431 protein-coding sequences.

## ANNOUNCEMENT

Bacteria belonging to the genus *Blautia* are common inhabitants of the human gastrointestinal tract (1) and have been associated, both positively and negatively, with a myriad of health conditions, including visceral fat accumulation (2), obesity and type 2 diabetes (3, 4), liver cirrhosis (5), and colorectal cancer (6). While their involvement in host health remains to be fully resolved, several studies have indicated that various *Blautia* species may provide beneficial health effects by alleviating inflammatory and metabolic diseases (7). The depletion of *Blautia luti*, along with *B. wexlerae*, in the feces of obese children has been implicated in metabolic complications such as insulin resistance (8). We used Oxford Nanopore Technologies (ONT) sequencing to generate a genome assembly of the type strain of *B. luti* (JCM 17040^T^ = DSM 14534^T^), formerly *Ruminococcus luti*, isolated from human feces (9).

Cells were obtained as L-dried cultures from the Japan Collection of Microorganisms (JCM) and cultured directly in peptone yeast extract glucose medium under an atmosphere of N_2_/CO_2_ [80/20 (vol/vol)] at 37°C. Genomic DNA was extracted using the MagAttract HMW DNA Kit (Qiagen) and prepared for sequencing using ONT’s Native Barcoding Kit 24 (SQK-NBD114.24, with Native Barcode 11), without fragmentation and following the manufacturer’s protocol. Sequencing was performed on an R10.4.1 flow cell using a GridION device, using default settings (translocation speed: 400 bp per second; sampling frequency: 4,000 per second). Bioinformatics procedures below used default settings unless specified. Bases were called with Dorado v0.2.1 (https://github.com/nanoporetech/dorado), using model dna_r10.4.1_e8.2_400bps_sup@v4.0.0. Reads were processed with Duplex Tools v0.3.1 (https://github.com/nanoporetech/duplex-tools) to identify and split chimeric and/or concatenated reads. Demultiplexing of co-sequenced libraries was performed using Guppy’s guppy_barcoder command (v6.4.6), with concurrent trimming of barcodes (options -enable_trim_barcodes -barcode_kits SQK-NBD114-24). Short (<1,000 bases) and low-quality (average base quality score <12) reads were removed using NanoFilt v2.8.0 (10). Filtered reads were then subsampled to a coverage of 150× using Filtlong v0.2.0 (https://github.com/rrwick/Filtlong), with option -mean_q_weight 10. A total of 111,114 reads (561,238,819 bases with an N50 of 6,439 bases) were retained and assembled using Flye v2.9.1 (11). This yielded a single contig marked as circular by Flye; the local alignment of the contig ends showed no overlap. The contig was rotated using SeqKit’s restart command (v2.2.0, 12) and polished with Medaka v1.7.3 (https://github.com/nanoporetech/medaka), specifying model r1041_e82_400bps_sup_v4.0.0. The genome assembly was inspected using CheckM’s lineage_wf (v1.1.3, 13), showing 99.4% completeness and 0.0% contamination. Annotation was performed with DDBJ’s Fast Annotation and Submission Tool v1.2.18 (14).

The genome of *B. luti* JCM 17040^T^ consists of a single circular chromosome with a length of 3,741,599 bp and a G + C content of 42.9%. Average nucleotide identity to a previously published draft genome sequence of *B. luti* JCM 17040^T^ (15) exceeded 99.99%, as estimated with Fastani v1.33 (16) using the draft genome as the query. Annotation predicted that the genome contains 21 ribosomal RNA genes, 60 transfer RNA genes, and 3,431 protein-coding sequences. We expect that the here-described complete genome sequence will enable a better understanding of the role of *B. luti* in the human gut.

## Data Availability

The genome sequence has been deposited in DDBJ/EMBL/GenBank under the accession number AP028156. Sequencing reads are available in DDBJ’s Sequence Read Archive (DRA) under accession number DRR462684.
